# The relationship between proteins of the mismatch repair system and the prognosis of prostate cancer: A systematic review

**DOI:** 10.1590/1678-4685-GMB-2025-0158

**Published:** 2026-05-01

**Authors:** Karina Serafim da Silva, Maria Clara Del Pintor Pasotti, Luiz Gustavo Neiva Reis Souza, Katia Ramos Moreira Leite, Sabrina T. Reis

**Affiliations:** ¹ Universidade de São Paulo, Faculdade de Medicina, Hospital das Clínicas HCFMUSP, Laboratório de Investigação Médica 55 (LIM55), São Paulo, SP, Brazil.; ² Faculdade de Medicina Atenas, 37900-380, Passos, MG, Brazil.; ³ Faculdade de Medicina de Marília, Marília, SP, Brazil.

**Keywords:** Prostate cancer, mismatch repair, microsatellite instability, systematic review

## Abstract

Prostate cancer (PCa) is a heterogeneous and prevalent neoplasm, traditionally stratified by PSA and Gleason Score. However, these biomarkers have prognostic limitations, driving the search for new molecular markers. Alterations in DNA mismatch repair (MMR) system proteins are associated with genomic instability, therapeutic resistance, and poorer clinical outcomes. Their relevance in PCa remains poorly understood, highlighting MMR status as a potential prognostic biomarker. This systematic review, following PRISMA 2020 guidelines, evaluated the relationship between MMR protein (MSH2, MSH6, MLH1, PMS2) expression and clinical-pathological outcomes in PCa. Ten studies assessing MMR protein expression in prostate adenocarcinoma samples were included. Risk of bias was assessed using the Newcastle-Ottawa Scale. Studies revealed heterogeneous MMR protein expression. Loss of MSH2 consistently correlated with poorer clinical outcomes, including biochemical recurrence, higher Gleason Scores, and perineural invasion. MSH6 was more prevalent in high-grade tumors, without clear prognostic association. Cytoplasmic MLH1 expression was linked to aggressive histological patterns; PMS2 results were conflicting. Two studies assessed Microsatellite Instability (MSI), correlating with MSH2 and PMS2. Overall, MMR protein alterations, particularly MSH2 loss, may indicate worse PCa prognosis. However, methodological heterogeneity and lack of standardization hinder definitive conclusions. Further studies, integrating MSI analyses are crucial to confirm their prognostic.

## Introduction

Prostate cancer (PCa) is the fourth most commonly diagnosed malignant neoplasm worldwide and the second most frequent cancer among men. Globally, PCa accounts for approximately 396,792 deaths per year (Bray et al., 2024). Although well-established biomarkers, such as PSA and Gleason score, have been incorporated into clinical practice for decades to aid in prognostic stratification, the highly variable clinical behavior of the disease underscores the need for novel biomarkers capable of predicting unfavorable outcomes more accurately. 

The MMR system plays a critical role in maintaining genomic stability. It repairs post-replicative single-strand DNA errors through protein complexes that act as heterodimers, recognizing and correcting incompatibilities between bases and insertion-deletion loops that arise during DNA replication (Pećina-Šlaus et al., 2020). At the molecular level, the MutSα heterodimeric complex (MSH2-MSH6) recognizes mismatched base pairs and binds to the mispaired bases to initiate repair. Subsequently, the MutLα complex (MLH1-PMS2) is recruited, which introduces single-strand breaks and promotes nucleotide excision to begin the resynthesis of the damaged DNA strand with the assistance of Exonuclease 1 (EXO1) and Ligase 1 (Awosika et al., 2025).

A distinctive feature of this pathway is that it preserves post-replicative repair integrity, particularly in DNA regions known as microsatellites, which are short, repetitive DNA sequences that are especially vulnerable to polymerase errors during replication. However, the occurrence of pathogenic alterations in one of the MMR complex subunits leading to inactivation and silencing of one of the pairs impairs efficient repair, resulting in the progressive accumulation of mismatches within these repetitive regions, ultimately giving rise to MSI, a phenotype directly linked to mismatch repair deficiency (dMMR) (Sedhom et al., 2019). In case, the dMMR and high MSI (dMMR/MSI-H) status serves as an important biomarker of response to immunotherapy with Immune Checkpoint Inhibitors (ICIs) in metastatic disease (Abida et al., 2019).

MMR pathway alterations have been described from two main perspectives: somatic inactivation, often associated with MLH1 promoter hypermethylation, and germline pathogenic variants associated with Lynch Syndrome (LS), an autosomal dominant condition caused by inherited inactivation of MMR genes. LS is typically linked to an increased risk of colorectal, endometrial, and gastric cancers, which exhibit dMMR and MSI-H (Pasanen et al., 2020; Venetis et al., 2025). Unlike these cancers, somatic alterations in PCa that lead to dMMR/MSI-H are observed more frequently in a hypermutated subtype, often characterized by complex structural rearrangements in MSH2 and MSH6 (Pritchard et al., 2014).

While dMMR/MSI-H is relatively common in several tumor types, its prevalence in PCa appears to be low. Current evidence suggests that dMMR/MSI-H occurs in only a small fraction of PCa cases (approximately 3-5%), more often in metastatic tumors, which display a hypermutated phenotype and are thus eligible for ICIs (Pritchard et al., 2014; Robinson et al., 2015; Abida et al., 2019).

Although less frequent in localized disease, there is growing clinical interest in exploring dMMR status as a potential biomarker of poor prognosis based solely on molecular features. However, the heterogeneous expression of these proteins in PCa has hindered advances in this area. One of the main challenges in establishing dMMR or MSI as a prognostic biomarker lies in identifying a method that is both sensitive and specific, yet cost-effective, for detecting these alterations. Currently, Next-Generation Sequencing (NGS) appears to be the gold standard for assessing MMR status in PCa, as it precisely characterizes the nature of the alteration (Hempelmann et al., 2018). Nevertheless, its high cost limits its feasibility in routine clinical practice. In this context, the present systematic review aims to synthesize evidence on the relationship between MMR pathway alterations in PCa and key prognostic factors, thereby guiding future research in this field.

## Material and Methods

### Study design

This systematic review was designed and reported in accordance with the Preferred Reporting Items for Systematic Reviews and Meta-Analyses (PRISMA) 2020 guidelines (Page et al., 2021). Eligible studies were those investigating the expression of the MMR proteins MSH2, MSH6, MLH1, and PMS2 in human PCa tissue samples, using immunohistochemistry as the detection method. A comprehensive literature search was conducted across PubMed, Web of Science, and Embase databases, restricted to studies published between 1995 and 2023. The search strategy included combinations of the following keywords and their relevant descriptors: “mismatch repair,” “MMR,” “microsatellite instability,” “prostate cancer,” “MSH2,” “MSH6,” “MLH1,” “PMS2,” and “immunohistochemistry,” using Boolean operators (AND, OR) to optimize sensitivity and specificity.

### Study selection

Study selection was conducted in two stages using the Rayyan platform (https://www.rayyan.ai). First, three independent reviewers screened articles based on titles and abstracts, with disagreements resolved in consultation with a fourth reviewer (the advisor). Next, the selected articles were reviewed in full text to assess compliance with the inclusion criteria. Any disagreements were resolved by consensus among the reviewers, or, if necessary, by the fourth reviewer (the advisor).

### Eligibility criteria (inclusion and exclusion)

Studies evaluating the expression of MSH2, MSH6, MLH1, and PMS2 proteins in PCa samples using immunohistochemistry were included. The studies aimed to assess the expression of these proteins in relation to clinical outcomes, including biochemical recurrence, overall survival, and disease-free survival. Literature reviews, case reports, conference abstracts, studies addressing Lynch syndrome, and studies analyzing other cancer types were excluded.

### Data extraction

A standardized form was used to collect relevant data, including author, year, location, objectives, sample characteristics, methodology, and main findings. Extracted variables included participant characteristics, MMR protein detection methods, protein expression results, and clinical outcomes.

### Quality assessment and risk of bias

The methodological quality of the included observational studies was assessed using the Newcastle-Ottawa Scale (NOS) (http://www.ohri.ca/programs/clinical_epidemiology/oxford.asp), as recommended for reviews involving cohort and case-control studies. This tool evaluates the risk of bias across three main domains: selection of study participants, comparability of groups, and assessment of exposure or outcome. The evaluation was performed independently by two reviewers, and any disagreements were resolved through consultation with a third reviewer to reach consensus.

### Data synthesis

Data were synthesized narratively and categorized into molecular and clinicopathological outcomes. Studies were grouped based on joint and individual analyses of MMR proteins and MSI, highlighting convergences and divergences in findings while considering study consistency and evaluated outcomes.

## Results

### Characteristics of the included studies

**Figure 1 -:**
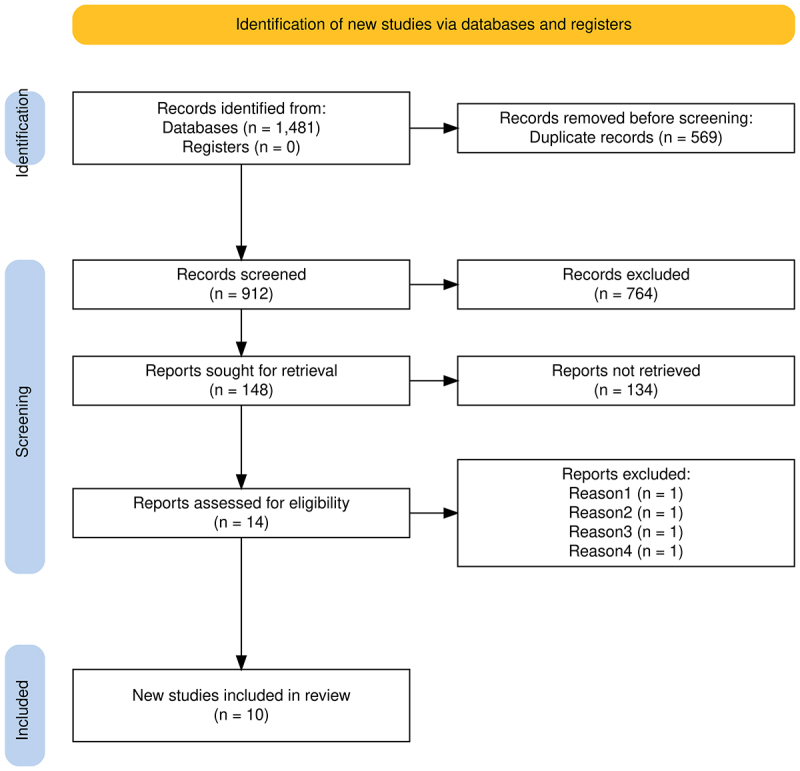
Flowchart of included studies based on PRISMA (2020) Guidelines for Systematic Reviews and Meta-Analyses.

The systematic search initially identified 1,481 records across the selected databases. After the manual removal of 569 duplicates, 912 unique records remained for screening. Title and abstract screening resulted in the exclusion of 764 records that did not meet the predefined eligibility criteria due to thematic irrelevance, absence of a direct relationship with the MMR system, focus on non-prostatic tumors, lack of available data on clinical outcomes, inadequate methodological designs, presence of LS, or inconsistency with the research question. Consequently, 148 studies were deemed potentially eligible and were assessed in full text. Following a detailed full-text evaluation, 134 articles were excluded for reasons including absence of the outcomes of interest, inadequate methodological description, incompatible study populations, or inability to extract the necessary information. The remaining 14 studies underwent an in-depth methodological quality assessment using standardized risk-of-bias tools, which led to the exclusion of four additional studies due to methodological weaknesses that could compromise the reliability of the synthesis. Ultimately, 10 studies met all eligibility and quality criteria and were included in the qualitative synthesis (Table 1). The complete study selection process is depicted in the PRISMA 2020 flow diagram (Page et al., 2021), presented in Figure 1.

**Table 1 -:** Description of included studies.

Author	Year	N	Evaluation Method	Expression Findings	Assessed Outcomes	Prognostic Associations	Age (years)	Follow-up
Norris *et al*.	2009	166	IHC: H-score	PMS2 present in 67% (111/166);	Biochemical recurrence, PSA, largest cancer volume, total tumor volume, prostate weight, and % Gleason 4/5	PMS2+: prostates with volume of the largest cancer and total tumor volume PMS2+: Associated with shorter recurrence-free survival in cancer. Associated with shorter recurrence-free survival among patients with a high percentage of Gleason pattern 4/5 as well.	5-76	5 years
Velasco *et al*.	2002	73	IHC: Semi-quantitative (intensity); MSI: 4-marker panel	hMSH2 moderate/high: 71% (52); low: 29% (21)	Biochemical recurrence	hMSH2−: low/absent staining associated with worse prognosis	51-76	49 months
Albero-González *et al*.	2019	200	IHC: Histoscore (negative: 0-10; positive: >10)	Low expression: MSH2 (8%), MLH1 (5%), PMS2 (7%); MSH6+: 85% (170/200)	GG; PSA recurrence	MSH6+: more frequent in GG5 (65%) than GG1-GG4 (39,4%) PMS2-: shorter PSA recurrence	-	1 to 212 months (mean: 102.5)
Guedes *et al*.	2017	1133	IHC: Qualitative;	Loss of MSH2: 1% (12/1133)	Gleason score	MSH2−: associated with Gleason score 5 (8%)	47-79	-
Norris *et al*.	2007	33	IHC: H-score; MSI: 5-marker panel	PMS2 present in 52% (17/33)	GG	PMS2+: associated with GG 3	41-53	-
Wilczak *et al*.	2017	11152	IHC: Semi-quantitative (negative to strong)	Low expression: MSH6 (15.4%), PMS2 (25.9%), MLH1 (18.5%), MSH2 (11.3%)	Pathological stage; Gleason score; Biochemical recurrence	MSH6, PMS2, MLH1+: associated with advanced stage, higher Gleason score, and recurrence	-	1-264 months (mean: 62.9; median: 50)
Sharma *et al*.	2020	220	IHC: Qualitative	Loss of ≥1 protein in 22.7% (50/220); MLH1 (6.36%), MSH2 (3.18%), MSH6 (4.54%), PMS2 (2.27%)	Preoperative PSA	Lower PSA in MMR+ group (5.76) vs MMR− (9.12)	42-78	3-116 months (mean: 48.2)
Jaworski *et al*.	2019	52	IHC: Semi-quantitative	MSH2: 8.8%, MSH6: 10%, MLH1: 30.2%, PMS2: 3.1%	Gleason score	MSH2+ (nuclear):	• Gleason score	•Gleason pattern	MSH2+ (cytoplasmic):	•Gleason score	MLH1+ (cytoplasmic):	•Gleason pattern	52-78	-
Folkmanis *et al*.	2022	92	IHC: Semi-quantitative	-	GG (1-5); Disease-free survival	MMR−: correlated with higher GG and shorter disease-free survival	43-85	Up to 5 years or until progression
Javeed *et al*.	2022	74	IHC: Qualitative	Loss: MSH2 (12.2%), MSH6 (2.7%), MLH1 (4.1%), PMS2 (5.4%)	Perineural invasion	MSH2−: more frequent in tumors with perineural invasion	50-98	6 months

Abbreviations: IHC - Immunohistochemistry; MSI - Microsatellite Instability; MMR - Mismatch Repair; GG - Grade Groups.

### Risk of bias assessment

The methodological quality and risk of bias of the included studies were assessed using the Newcastle-Ottawa Scale (NOS), summarized in Table 2. This tool, recommended by the Cochrane Collaboration for observational and prognostic studies (http://www.ohri.ca/programs/clinical_epidemiology/oxford.asp), evaluates studies across three domains: selection of participants (maximum score: 4), comparability of groups (maximum score: 2), and outcome assessment (maximum score: 3). Studies scoring 7-9 were classified as low risk of bias, 4-6 as moderate, and 0-3 as high.

**Table 2 -:** Assessment of study quality and risk of bias according to the NOS guidelines.

Article (Year)	Selection (max 4★)	Comparability (max 2★)	Outcome/Exposure (max 3★)	Total (★)
Norris et al. (2009)	4	2	3	9
Velasco et al. (2002)	3	2	2	7
Albero-González et al. (2019)	4	1	2	6
Guedes et al. (2017)	4	2	2	8
Norris et al. (2007)	4	2	2	8
Wilczak et al. (2017)	4	2	2	8
Sharma et al. (2020)	3	2	2	7
Jaworski et al. (2019)	3	2	2	7
Bansal et al. (2012)	3	1	2	6
Folkmanis et al. (2022)	4	2	2	8
Javeed et al. (2022)	4	2	2	8

### MMR proteins and association with clinicopathological outcomes

*Overall MMR (MSH2, MSH6, MLH1, and PMS2)*

*Combined evaluation of MSH2, MSH6, MLH1, and PMS2 has revealed associations with several clinicopathological features, particularly disease-free survival and Grade Groups (GG) (1-5). Loss of these proteins correlated with higher GG and shorter disease-free survival (22 months vs. 66 months) (* *Folkmanis* et al., 2022). In contrast, preserved expression of these proteins was linked to lower PSA levels (5.76 vs. 9.12) (Sharma et al., 2020). Notably, when MSH2 was excluded from the analysis, the presence of MSH6, MLH1, and PMS2 remained significantly associated with adverse prognostic factors, including advanced pathological stage, higher Gleason score, lymph node metastasis, and early biochemical recurrence (Wilczak et al., 2017).

*MSH2*

Three studies specifically investigated the prognostic significance of MSH2 loss (Velasco et al., 2002, Guedes et al., 2017, Javeed et al., 2022). Despite some variability, all reported that MSH2 loss was associated with unfavorable outcomes. Absence of MSH2 correlated with higher risk of biochemical recurrence and shorter disease-free survival (Velasco *et al*., 2002), was more frequent in Gleason pattern 5 (Guedes *et al*., 2017), and was linked to perineural invasion (Javeed *et al*., 2022). Conversely, one study documented the presence of MSH2 and observed that nuclear and cytoplasmic staining was more frequent in tumors with Gleason score ≥7, with only nuclear staining correlating significantly with Gleason pattern (3-5) (Jaworski et al., 2019).

*MSH6*

Regarding MSH6, the dimerization partner of MSH2, no studies identified its loss as a predictive of clinical outcomes. Nonetheless, isolated MSH6 presence was reported more frequently in GG5 (65%) compared to GG1-GG4 (39.4%) (Albero-González et al., 2019).

*MLH1*

Isolated loss of MLH1 was not observed in the studies included. However, cytoplasmic MLH1 presence was detected and positively correlated with higher Gleason patterns (3-5) (Jaworski et al., 2019).

*PMS2*

PMS2 exhibited heterogeneity in both expression patterns and associated outcomes. One study reported PMS2 loss as associated with shorter PSA recurrence time (Albero-González et al., 2019). In contrast, PMS2 presence was associated with Gleason Grade 3, larger cancer volume, and greater total tumor volume (Norris et al., 2007, 2009). Intriguingly, PMS2 presence was also linked to shorter recurrence-free survival in patients with a high percentage of Gleason 4/5 patterns (Norris *et al*., 2009).

### MSI and associated molecular profile

Given the clinical relevance of MSI and its interplay with MMR status, all studies addressing these markers were analyzed for potential associations. Of the 10 studies, only two assessed MSI explicitly (Velasco et al., 2002, Norris et al., 2007). Tumors with elevated PMS2 expression demonstrated instability, whereas concomitant elevation of its dimer partner MLH1 abrogated this instability (Norris *et al*., 2007). It has also been reported that, in clinically localized prostate carcinomas, approximately 60% of tumors exhibiting MSH2 loss by immunohistochemistry also displayed MSI (Velasco *et al*., 2002).

## Discussion

This study aimed to synthesize evidence on alterations in MMR proteins in PCa and their association with prognostic factors. Our analysis echoes a critical question raised in the literature: the true clinical significance of MMR protein alterations in PCa and whether they indeed correlate with worse outcomes. Initially, we intended to focus solely on the dMMR phenotype, but as we delved into the literature, it became clear that limiting our scope to deficiency alone would overlook relevant findings. Several studies reported variable expression of MSH2 and PMS2 associated with both adverse and favorable prognoses (Velasco et al., 2002, Norris et al., 2007, 2009, Guedes et al., 2017, Albero-González et al., 2019, Jaworski et al., 2019, Javeed et al., 2022). Findings related to MLH1 and MSH6 were less frequent and generally limited to the predictive value of their isolated presence across different prognostic parameters (Albero-González *et al*., 2019, Jaworski *et al*., 2019).

A critical biological aspect to consider is that these proteins operate as heterodimers to fulfill their DNA repair function. MSH2 and MSH6 recognize mismatches and initiate base excision, while MLH1 and PMS2 reinstate the correct nucleotides. Acting at microsatellite regions, the absence of any component disrupts the pathway, rendering repair ineffective (Iyer et al., 2006). Notably, only two of the 10 studies evaluated MSI status explicitly (Velasco et al., 2002, Norris et al., 2007). This is particularly striking since Norris *et al*. demonstrated an association between PMS2 expression and MSI, which disappeared when PMS2 was co-expressed with MLH1 (Norris *et al*., 2007). These findings highlight the relevance of MSI testing as a confirmatory marker of dMMR, especially because loss-of-function variants may not always result in loss of protein expression. Given the lack of a validated MSI panel for PCa, it is worth noting that prostate tumors exhibit higher instability in dinucleotide repeats (Perinchery et al., 2000), suggesting that the panel proposed by the NCI (Boland et al., 1998) could be an alternative to improve consistency in MMR status assessment, either for dMMR confirmation or as a complementary test.

Another important challenge lies in the absence of a gold-standard test for assessing the molecular MMR profile in PCa. Although immunohistochemistry remains the preferred method due to its feasibility and good performance, substantial methodological and clinical heterogeneity persists across studies. Quantification methods varied widely, ranging from qualitative assessments to semi-quantitative scoring based on staining intensity or composite indices. Furthermore, definitions of clinical outcomes also lacked uniformity: aside from the nearly universal use of Gleason score, other prognostic markers were inconsistently applied.

We acknowledge the limitations of the current evidence. Unlike tumors where dMMR/MSI is prevalent and routinely screened, such as colorectal and endometrial cancers, the prevalence in PCa is low (~5%), raising questions about the utility of routine screening. Moreover, the literature lacks sufficient evidence to clearly define which PCa subgroups might benefit most from dMMR and MSI testing.

In designing this review, we deliberately avoided bias by not restricting our analysis to loss of protein expression alone. Our findings suggest that both the presence and absence of these proteins may carry clinical implications, and the lack of a gold-standard assessment method warrants caution before proposing them as prognostic markers. We believe that expanding the evidence base through large, well-designed cohort studies with multivariate analyses could substantially improve understanding of the clinical relevance of MMR proteins in PCa.

## Conclusion

The evidence synthesized in this systematic review supports a relevant association between the MMR system and PCa progression. Among the ten included studies, loss of MSH2 expression emerged as the alteration most consistently associated with adverse clinical outcomes. Three of the four studies that specifically evaluated this protein linked its absence to higher Gleason scores, early biochemical recurrence, and perineural invasion, reinforcing its potential role in tumor aggressiveness. In contrast, the findings related to MSH6, MLH1, and PMS2 were limited and inconsistent, likely to reflect substantial methodological heterogeneity, as these proteins were not uniformly assessed across studies.

**Figure 2 -:**
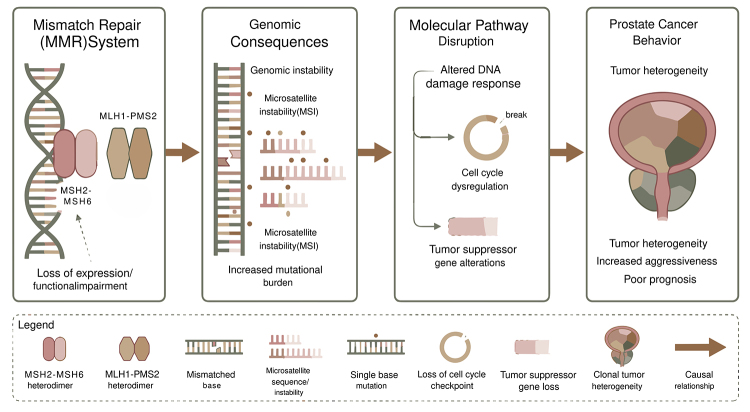
Conceptual model summarizing the impact of MMR system alterations on PCa behavior. Loss of MMR function impairs the correction of single-strand DNA mismatches, resulting in genomic instability, MSI, and increased mutational burden. These alterations disrupt DNA damage response pathways, cell cycle control, and tumor suppressive pathways, ultimately contributing to tumor heterogeneity, increased aggressiveness, and poor prognosis in PCa.

From a mechanistic perspective, the MMR system comprises four core proteins that function as obligate heterodimers (MSH2-MSH6 and MLH1-PMS2). Functional impairment of any single component is sufficient to destabilize the complex, leading to genomic instability and failure of post-replicative mismatch correction, with consequent accumulation of secondary mutations in microsatellite regions. In PCa, MMR inactivation may promote tumor heterogeneity by enabling genetic alterations in key suppressor genes involved in cell cycle regulation, DNA damage response, and apoptosis (Figure 2). Nevertheless, unlike advanced and metastatic disease, where dMMR/MSI-H status has clear clinical implications for predicting response to ICIs, the relevance of dMMR in localized PCa remains constrained by its low prevalence, lack of standardized assessment methods, and the limited sample sizes of available studies, which collectively restrict its prognostic applicability.

Overall, these findings underscore the need for future studies based on larger, well-characterized cohorts, standardized immunohistochemistry and MSI assessment protocols, and highly sensitive molecular techniques such as NGS. Such efforts are crucial for clarifying the true prognostic significance of MMR alterations in localized PCa and aimed at optimal strategies for their detection. In this context, loss of MMR protein expression, particularly MSH2, should be interpreted cautiously and not regarded as an incidental finding, given its association with aggressive disease behavior.

## Data Availability

The complete dataset supporting the findings of this study is provided within the article itself.
